# A Review of Radiological Investigations in Cases of Acute Appendicitis in a Tertiary Care Hospital

**DOI:** 10.7759/cureus.36916

**Published:** 2023-03-30

**Authors:** Dalal R Hubail

**Affiliations:** 1 General Surgery, Salmaniya Medical Complex, Manama, BHR

**Keywords:** mri, ultrasound, ct scan, radiology, acute appendicitis

## Abstract

Introduction: Acute appendicitis is the most common non-traumatic surgical emergency and early diagnosis and management are crucial to decrease morbidity and mortality. There is a variety of scoring systems and radiological investigations that have been used in the diagnosis of acute appendicitis. Hence, the aim of this study is to assess the diagnostic approach in patients with suspected appendicitis in a tertiary care hospital, focusing on the radiological burden.

Methods:This is a cross-sectional study reviewing the electronic and manual medical records of all adult patients admitted with the diagnosis of acute appendicitis between January 2018 and December 2018 in Salmaniya Medical Complex, Manama, Bahrain. A review of the method of diagnosis (clinical, ultrasound, computed tomography, or others) was done, with a comparison to histopathological results in those that underwent surgical intervention to determine sensitivity and specificity.

Results: In the study period, 488 patients were admitted with acute appendicitis; out of these, 461 underwent surgical intervention. A total of 66 CT scans and 148 ultrasounds were conducted for these patients, out of which 57% of ultrasounds and 86% of CT scans accurately diagnosed acute appendicitis based on histopathological diagnosis, resulting in a sensitivity of 65% and 92%, respectively, and a specificity of 56% and 25%, respectively.

Conclusion: The most accurate method of diagnosis of acute appendicitis (highest sensitivity) is CT scanning. However, a prospective study with a detailed assessment of complications of appendicitis is recommended.

## Introduction

Acute appendicitis is the most common non-traumatic surgical emergency worldwide, with an incidence of 100-150 per 100,000 person-years [1]. It is associated with significant morbidity, mortality, and healthcare costs; however, advancements in diagnosis and management have shown a 46% reduction in mortality from acute appendicitis [1]. Therefore, early diagnosis and management are crucial to mitigate these risks.

Diagnosis of appendicitis is divided into two aspects: accurate diagnosis followed by stratification into uncomplicated versus complicated disease [2,3]. This is integral to the management of this common surgical condition as the approach to the patient differs based on the complexity of the condition [2,3]. Therefore, in addition to history, physical examination, laboratory tests, and different scoring systems, radiological imaging has become an integral part of the management of acute appendicitis. The two main radiological investigations used in appendicitis are abdominal ultrasonography (US) and computed tomography (CT) [2,3].

Initially, ultrasound was considered a reliable imaging study for appendicitis; however, an accuracy assessment showed that its sensitivity and specificity are moderate at best 59-78% and 73-88%, respectively, mainly due to it being operator dependent [4,5]. On the other hand, CT scanning has emerged as the imaging study of choice for appendicitis owing to its high sensitivity (93-96%) and specificity (92-95%); however, there are concerns regarding the radiation exposure associated with CT scanning [2,6]. Moreover, CT scanning is highly recommended in older individuals to identify malignancies presenting as acute appendicitis [2,6].

Furthermore, studies have evaluated the use of magnetic resonance imaging (MRI), especially in pregnant females, since it eliminates the risk associated with radiation with a sensitivity of 87-98% and specificity of 96-98% [7]. However, access to MRI facilities especially after hours is challenging, in addition to the fact that MRI has a lower diagnostic accuracy (sensitivity of 39-73%) for complicated appendicitis compared with CT scanning [7,8].

Therefore, the aim of this study is to assess the diagnostic approach to acute appendicitis in a tertiary hospital in Bahrain focusing on the use and accuracy of advanced radiological imaging.

## Materials and methods

Study design and setting

This is a single-center, retrospective, cross-sectional study spanning from January 1 to December 31, 2018, set in Salmaniya Medical Complex, the main public hospital in the Kingdom of Bahrain, located in Manama. The study looked at cases admitted with the diagnosis of acute appendicitis to determine the method of diagnosis, focusing on the different radiological studies (abdominal and chest x-rays, US of the abdomen, CT of the abdomen, MRI). Furthermore, the use of advanced radiological imaging (US, CT, and MRI) was compared to the clinical diagnosis of acute appendicitis (typical presentation +/- baseline plain x-rays). Ethical Approval was obtained from the Research Committee for Government Hospitals for this study (approval number: 9140223).

Participants

**Figure 1:**
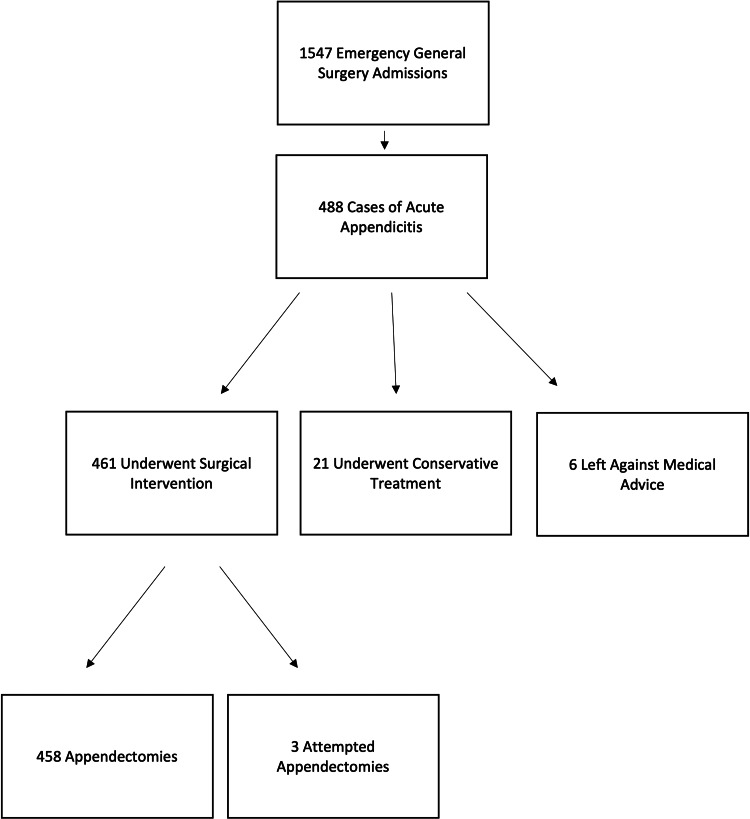
Flow Diagram

Within the study period, 488 cases were admitted with a diagnosis of acute appendicitis, fulfilling the inclusion and exclusion criteria below. Figure 1 shows the participant flow diagram. 

Inclusion and exclusion criteria

Patients with an admitting diagnosis of acute appendicitis (including both complicated and non-complicated appendicitis) aged 14 years and above were included. The following were the exclusion criteria: Patients admitted as abdominal pain for observation or with a different initial diagnosis that was later found to have appendicitis, Pediatric age group (i.e. less than 14 years), and those with data missing (such as from system error or inability to access manual files).

Sample size and data collection

The sample size was determined based on convenience sampling of one year, with an estimate of 400 patients. The data was collected from both electronic and manual medical records to ensure maximum efficacy. The information collected for each patient included demographic data, method of diagnosis (clinical versus advanced radiological), type of radiological imaging used, method of treatment (surgical versus conservative treatment), and the histopathological results of the appendectomy specimens (if applicable). The radiological studies used were abdominal x-rays, chest x-rays, US, CT, MRI, or others, with each designated one of four categories as detailed in Table 1.

**Table 1:** Designated Categories for the Radiological Investigations

Description	Definition
Diagnostic positive	A clear diagnosis of acute appendicitis was reached
Non-diagnostic positive	A positive finding was noted in the imaging, but did not provide a final diagnosis (such as free fluid in the right iliac fossa but appendix was not visualized)
Diagnostic negative	A negative radiological scan that clearly excludes acute appendicitis and provides an alternative diagnosis
Non-diagnostic negative	A radiological scan that doesn’t exclude appendicitis or provide an alternative diagnosis (patient may need further imaging or be managed based on clinical presentation)

Data analysis

The data were analyzed using Excel (Microsoft Corporation, Redmond, Washington, United States) to determine the demographics of the study population, in addition to calculating the following: total number of cases of acute appendicitis (complicated and non-complicated), total number that underwent imaging, and total number of each imaging study and their diagnostic yield. For CT and US, the results were compared with the final histopathological diagnosis to calculate the percentage of accurate diagnoses by each imaging modality; hence, calculating the sensitivity and specificity. 

## Results

Demographics

The study spanned one year from January 1 to December 31, 2018, with an evaluation of emergency general surgery admissions in patients aged 14 and above (n=1547). Of these, 488 (31.5%) were cases of acute appendicitis (382 males, 106 females). The ages of these patients ranged between 14 to 77 years old, with the majority (76%) between 20 to 39 years (n=371) with a mean age at diagnosis being 30.5 years (± 9 SD). 

**Figure 2:**
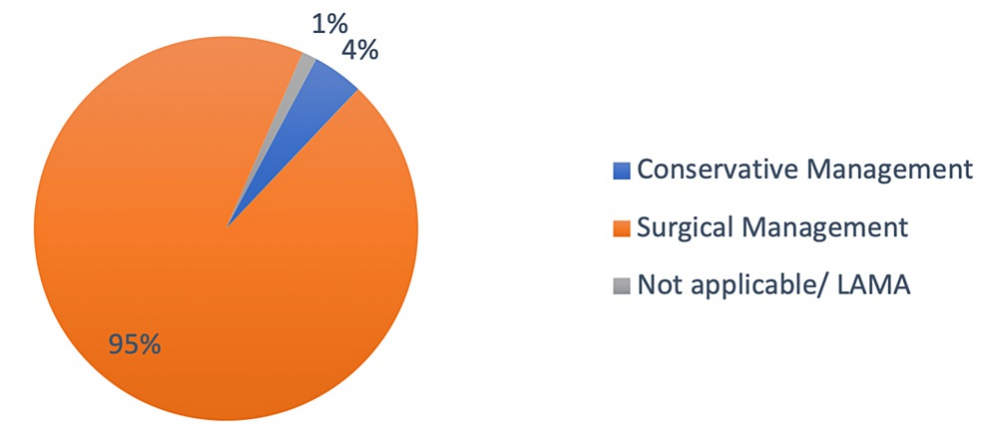
Types of Interventions in Acute Appendicitis LAMA: left against medical advice

In addition to the diagnosis of acute appendicitis, the abovementioned cases included one case of appendicular stumpitis and 27 cases of appendicular mass. Of the total number of cases, 461 (94%) underwent surgical intervention (including three attempted appendectomies for appendicular masses that were not diagnosed preoperatively), six left against medical advice (one was readmitted and operated), and the remaining were treated conservatively for stumpitis, appendicular mass, and patient preference (due to the cardiac risk associated with surgery). Details are noted in Table 2 and Figure 2. 

**Table 2:** Types of Interventions in Cases of Acute Appendicitis *Including one with Meckel’s diverticulectomy **seven out of these were reported as diagnostic laparoscopy and appendectomy

Type of Intervention	Total Number	Percentage
Open Appendectomy (Lanz)	353*	72%
Attempted Open Appendectomy	2	0.4%
Laparotomy + Appendectomy	2	0.4%
Laparoscopic Appendectomy	100**	20%
Diagnostic Laparoscopy + Attempted Appendectomy	2	0.4%
Laparoscopic converted to Open Appendectomy	2	0.4%
Conservative	21	4%
Left Against Medical Advice (LAMA)	6	1%

Radiology burden in appendicitis 

**Figure 3:**
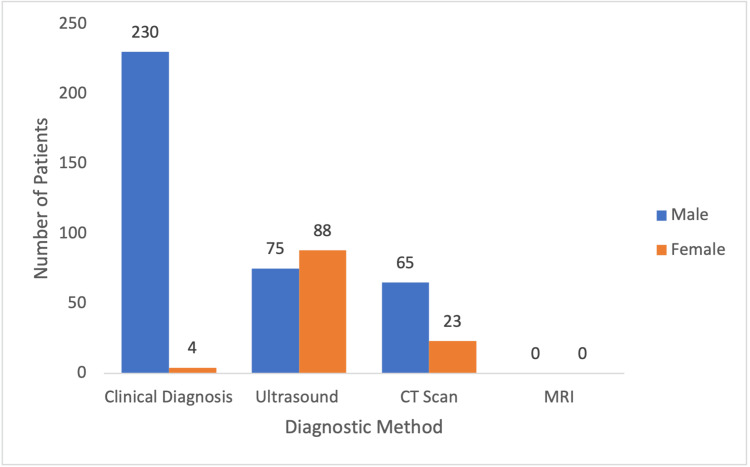
Diagnostic Approach in Acute Appendicitis by Gender

Out of the 488 cases of appendicitis admitted in the study period, 483 (99%) underwent a form of radiological investigation, ranging from baseline plain x-rays (chest and/or abdomen), CT of the abdomen and US, or a combination of these. Of these patients, 378 were males and 105 were females. Table 3 and Figure 3 detail the distribution of the patients between clinical diagnosis (typical presentation +/- baseline plain x-rays) and advanced radiological imaging (US, CT, and MRI) according to age and gender.

**Table 3:** Diagnostic Approach in Acute Appendicitis

	<40 years		>40 years		Total Number
	Male	Female	Male	Female	
Clinical Diagnosis	211 (90%)	3 (1%)	19 (8%)	1 (0.4%)	234
Ultrasound	65 (40%)	81 (50%)	11 (7%)	6 (3%)	163
CT Scan	44 (50%)	17 (19%)	21 (24%)	6 (7%)	88
MRI	0	0	0	0	0

The majority (n=465; 95%) started with basic plain x-rays (either chest, abdomen, or both) as a baseline imaging rather than a diagnostic one, with 298 chest x-rays and 454 abdominal x-rays. All of the plain x-rays were non-diagnostic, so the diagnosis was based on either typical presentation or further advanced imaging. 

Furthermore, 163 patients underwent US of the abdomen to assess their symptoms (87 females and 76 males). The majority 101 (61%) were diagnostic positive and showed acute appendicitis. However, eight were diagnostic negative, 27 were non-diagnostic positive, and 27 were non-diagnostic negative. On the other hand, CT abdomen was conducted for a total of 88 patients (65 males versus 23 females). Out of these, 80 (91%) were diagnostic positive (while six non-diagnostic positive and two were non-diagnostic negative). Details are described in Table 4. 

**Table 4:** Diagnostic Yield of Ultrasound and CT Scan

	Diagnostic positive			Diagnostic negative		Non-diagnostic positive		Non-diagnostic negative	
	Male	Female		Male	Female	Male	Female	Male	Female
Ultrasound Abdomen	52		49	2	6	10	17	12	15
CT Scan Abdomen/Pelvis	63		17	0	0	1	5	1	1

Moreover, of the above-discussed patients, 29 underwent both US and CT scans (16 males vs 13 females). The initial imaging modality in all of them was US, with an inconclusive result; therefore, the decision was made to proceed to CT scanning. Out of these CT scans, 26 were diagnostic positive, while one was non-diagnostic negative, and two were non-diagnostic positive.

Out of the total number of patients, 41 underwent imaging (either US or CT) in another hospital prior to presenting to our center. These included 35 US scans of the abdomen, while six were CT scans. From these, eight patients underwent repeat imaging at our center. Of these eight patients, one underwent repeat US, five underwent CT scanning, one proceeded to both US and CT scanning, and the last patient underwent a repeat CT scan at our center as appendicular mass was suspected.

Comparison of imaging and histopathological results 

Of our total cases of acute appendicitis, 461 underwent surgical intervention. Of these, 458 underwent appendectomy and three underwent attempted appendectomy (for appendicular mass), as detailed in Table 1. From these, 46 underwent CT scanning, 128 underwent US, 20 underwent both US and CT in our center, 31 underwent US (five repeated in our center: three CT and 2 US) and four underwent CT scan prior to presenting to our center. Finally, the remaining patients (232) were diagnosed clinically based on typical presentation. 

**Figure 4:**
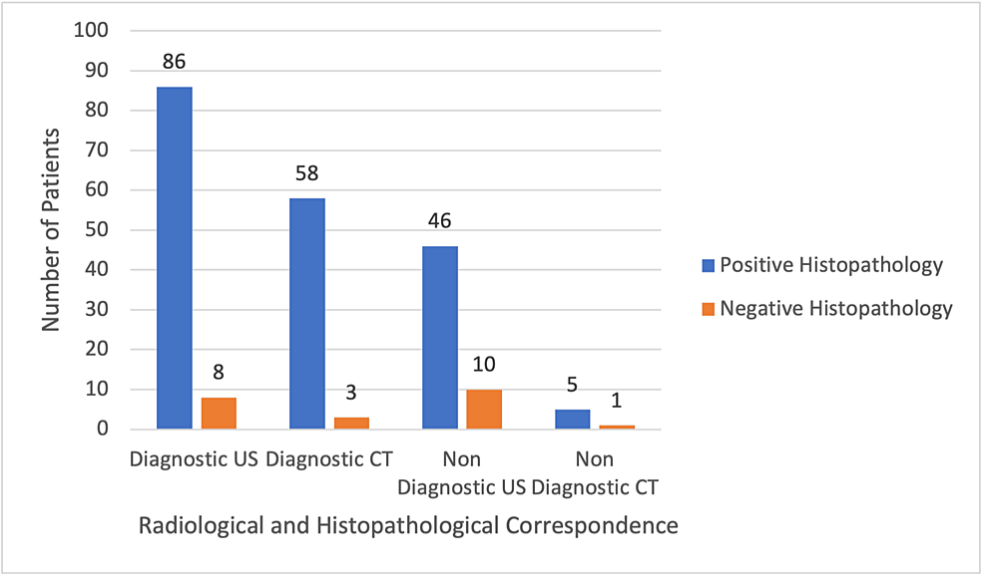
Accuracy of Advanced Imaging Based on Histopathological Diagnosis

As shown in Table 5 and Figure 4, 86 (57%) cases were correctly diagnosed by ultrasound, while eight were found to have a normal appendix on histopathological examination but were diagnosed as acute appendicitis by ultrasound. Hence, this shows that ultrasound has a sensitivity of 65% for the diagnosis of acute appendicitis, with a specificity of 56%.

**Table 5:** Ultrasound versus Histopathological Diagnosis of Acute Appendicitis

	Positive histopathology	Negative histopathology
Diagnostic Ultrasound	86	8
Non-Diagnostic Ultrasound	46	10

On the other hand, 58 (87%) cases were accurately diagnosed by CT scanning, while three were diagnosed as appendicitis but histopathological examination showed a normal appendix (further details are described in Table 6 and Figure 4). Therefore, this shows that CT scanning has a sensitivity of 92% for the diagnosis of acute appendicitis, with a specificity of 25%. 

**Table 6:** CT Scan versus Histopathological Diagnosis of Acute Appendicitis

	Positive histopathology	Negative histopathology
Diagnostic CT Scan	58	3
Non-Diagnostic CT Scan	5	1

Finally, from the 234 cases diagnosed clinically, 213 (91%) had positive histopathology for appendicitis, while 17 had a normal appendix. 

## Discussion

A vast array of research has been conducted to ease the diagnosis of different diseases, especially acute appendicitis, being the most common surgical emergency. Many studies have been conducted to assess the approach to patients suspected of having acute appendicitis with a combination of clinical assessment (using scoring systems), laboratory investigations, and imaging studies to determine the optimal diagnostic method. 

Debate is ongoing on the need for radiological imaging to diagnose appendicitis before surgery and the optimal type of radiological investigations. The following are the updated guidelines from the World Society of Emergency Surgery 2020 regarding the diagnosis of acute appendicitis: (1) Patients with intermediate risk of appendicitis based on clinical judgment and scoring systems should undergo an imaging study to reach a final diagnosis, (2) Patients younger than 40 years with a high risk of acute appendicitis based on presentation and scoring systems can proceed with diagnostic laparoscopy rather than imaging, and (3) The initial recommended imaging study in cases of suspected appendicitis that require radiological confirmation is US, followed by low-dose CT [9]. 

The abovementioned recommendations are in line with the data from our center, where male patients below the age of 40 with a typical presentation of appendicitis proceed to surgery without the need for advanced imaging. However, females, older age, obesity, and patients with atypical presentations were investigated further. Most patients with obesity, atypical presentations, and at an older age underwent CT scans, while younger patients and females with typical presentations underwent first-line US imaging. A study by Sauvain et al. corroborated this by determining that US imaging is usually inconclusive in obese patients, and that CT is the optimal imaging for appendicitis in this patient population [10]. Further studies investigated reasons that increase the chance of an inconclusive US, which included obesity [11,12] amongst other factors, such as advanced age [11,12], unusual location of the appendix, and complicated appendicitis [11]. 

Moreover, many of the patients who underwent US needed further imaging with CT scan to reach a final diagnosis. This is likely due to US being highly operator dependent. This result from our center aligns with the data in the literature, where CT has been found to be superior in diagnosing appendicitis. This was clearly noted in a Cochrane review, where it was found that CT scan has high sensitivity and specificity for diagnosing acute appendicitis; however, unenhanced CT scan was noted to have lower sensitivity than contrast-enhanced imaging. This has been further elucidated by Karul et al., who concluded that appendiceal wall thickening is enhanced with intravenous contrast, hence, making diagnosis easier [13]. In addition, studies have shown that negative appendectomy rates are low with liberal use of CT scanning; however, there should be a standardized definition to diagnose appendicitis on CT [14]. Although CT was found in the literature to have a high sensitivity and specificity for the diagnosis of acute appendicitis, many guidelines recommend starting with US due to the decreased radiation exposure and cost-effectiveness [15]. Alternatively, a study by Sippola et al. concluded that low-dose CT scan is a viable alternative with a similar diagnostic yield but lower radiation exposure [16]. Furthermore, CT scanning has been recognized as the tool of choice for assessing complications of acute appendicitis, from abscess formation, perforation, and gangrene to portal vein thrombosis and liver abscesses [15].

Furthermore, MRI has emerged as a valid option in cases where radiation needs to be avoided, namely, in pediatrics [17] and pregnant females [18]. However, due to the cost and logistics of accessing MRI facilities after official working hours, its use is limited [15]. In our study group, we did not note any patient that proceeded to MRI, mainly because it focused on adults rather than pediatrics in addition to the lack of pregnant females with appendicitis encountered in our study period. 

It is important to note that most patients underwent plain x-rays of the chest, abdomen, or both when presenting with symptoms suspicious of acute appendicitis. However, all of them were non-conclusive, and either the patient proceeded to surgery based on typical symptoms or required further imaging. A review by Teng et al. addressed the use of plain x-rays in acute appendicitis, where seeing air under the diaphragm on a chest x-ray with perforated appendicitis was noted to be a rare occasion, and abdominal x-rays showing an appendicolith or a mass in the right lower quadrant were almost non-existent [15].

An important point to mention is that the increased use of radiological imaging for the diagnosis of appendicitis comes at an equivalent increase in costs. Therefore, it was found that the use of imaging varied based on the country’s income, as well as within the country based on the characteristics of the patients (as detailed above) and hospital capabilities [9,15]. In our center, both US and CT scanning are easily accessible throughout the day. However, limitations do occur when MRI is needed, mainly, with pregnant females.

Finally, limitations of the study include its retrospective nature, which influences the data based on the accuracy of the medical records. Furthermore, a determination of who performed the US or reviewed the CT might shed some light on the varying accuracy of the results, as both can vary based on the experience of the radiologist with the tendency of emergency imaging being conducted by less experienced junior radiologists. In addition, further subcategorizing the diagnosis of appendicitis into complicated and uncomplicated was not investigated. Moreover, the specificity calculations may be influenced by the fact that patients don't usually undergo appendectomy if advanced imaging rules out acute appendicitis.

## Conclusions

Diagnostic imaging plays a crucial role in the management of patients with suspected acute appendicitis. The imaging modality of choice depends on local availability in addition to the individual characteristics of each patient. Overall, CT has been found to be the most sensitive and specific, which is supported by the data from our center. However, in certain populations such as children and pregnant females, other imaging modalities have been favored, such as US and MRI, respectively.

A further prospective study could be conducted to corroborate our results. In addition, it can also evaluate the diagnostic accuracy of specific complications of appendicitis (such as mass formation and perforation) using imaging modalities available in our center.
